# Stem Cell-Derived Extracellular Vesicles as Immunomodulatory Therapeutics

**DOI:** 10.1155/2019/5126156

**Published:** 2019-02-26

**Authors:** Yoojin Seo, Hyung-Sik Kim, In-Sun Hong

**Affiliations:** ^1^Institute of Translational Dental Sciences, School of Dentistry, Pusan National University, Yangsan 50612, Republic of Korea; ^2^Department of Life Science in Dentistry, School of Dentistry, Pusan National University, Yangsan 50612, Republic of Korea; ^3^Laboratory of Stem Cell Research, Lee Gil Ya Cancer and Diabetes Institute, Gachon University, Incheon 21999, Republic of Korea; ^4^Department of Molecular Medicine, School of Medicine, Gachon University, Incheon 21999, Republic of Korea

## Abstract

Mesenchymal stem cells (MSCs) have been reported to possess regulatory functions on immune cells which make them alternative therapeutics for the treatment of inflammatory and autoimmune diseases. The interaction between MSCs and immune cells through paracrine factors might be crucial for these immunomodulatory effects of MSCs. Extracellular vesicles (EVs) are defined as bilayer membrane structures including exosomes and microvesicles which contain bioactive paracrine molecules affecting the characteristics of target cells. Recently, several studies have revealed that EVs derived from MSCs (MSC-EVs) can reproduce similar therapeutic impacts of parent MSCs; MSC-EVs could regulate proliferation, maturation, polarization, and migration of various immune effector cells and modulate the immune microenvironment depending on the context by delivering inflammatory cytokines, transcription factors, and microRNAs. Therefore, MSC-EVs can be applied as novel and promising tools for the treatment of immune-related disorders to overcome the limitations of conventional cell therapy regarding efficacy and toxicity issues. In this review, we will discuss current insights regarding the major outcomes in the evaluation of MSC-EV function against inflammatory disease models, as well as immune cells.

## 1. Introduction

Mesenchymal stem cells (MSCs), which can be alternatively defined as multipotent stromal cells, can self-renew and differentiate into various cell types, such as osteocytes, adipocytes, chondrocytes, cardiomyocytes, fibroblasts, and endothelial cells [1–3]. MSCs reside throughout the body and can be obtained from a variety of tissues including bone marrow, adipose tissue, gingiva, dental pulp, and tonsil, as well as from the immature tissues including amniotic fluid, placenta, and umbilical cord or cord blood. In addition, MSCs differentiated from induced pluripotent stem cells (iPSCs) have been studied due to their superior self-renewal ability compared to conventional MSCs, although their safety and efficacy concerns are still challenging [4]. Depending upon their origin, MSCs present different physiological properties such as proliferative and differentiation capacity [5]; in general, however, many reports have supported that MSCs critically contribute to the maintenance of the microenvironment for tissue homeostasis and the tissue regeneration and remodelling upon injury. Moreover, MSCs have been known to regulate the functions of immune cell from both innate immunity and adaptive immunity, that is, MSCs can suppress the proliferation, differentiation, and activation of T cells, B cells, macrophages, dendritic cells, and natural killer (NK) cells, especially when these immune cell responses are excessive [6–9]. This immunomodulatory effect of MSCs on immune cells is exerted by the secretion of soluble factors such as prostaglandin-E_2_ (PGE_2_), indoleamine 2,3-dioxygenase-1 (IDO-1), nitric oxide (NO), transforming growth factor- (TGF-) *β*1, hepatocyte growth factor (HGF), and interleukin- (IL-) 10 [8, 10–15]. Indeed, this immunomodulatory ability of MSCs has been investigated for the treatment of various immune-related disorders, including inflammatory bowel disease, collagen-induced arthritis, sepsis, graft-versus-host disease, multiple sclerosis, and type I diabetes [16–22]. More recently, several studies have reported the beneficial outcomes of MSC application in allergic diseases such as asthma and dermatitis [23–30].

Extracellular vesicles (EVs) are bilayer membrane structures transferring bioactive components, including proteins, lipids, and coding and noncoding RNAs [31–33]. The best-studied EVs can be classified into exosomes and microvesicles according to their respective sizes, shapes, biogenesis, origins, and composition. Exosomes are homogenous in their size ranging from 40 to 200 nm and are generated through the invagination of the endosomal membrane of multivesicular bodies (MVBs), followed by fusion of MVBs with the plasma membrane and subsequent exocytosis. On the other hand, microvesicles are relatively heterogeneous in size ranging from 50 to 1000 nm and are released through the direct shedding or budding from the plasma membrane [33, 34]. Since no specific markers for the discrimination of exosomes and microvesicles are available, so far, all vesicles obtained experimentally are named as EVs [35, 36]. The released EVs mediate cell-to-cell communication by exchanging bioactive molecules with neighboring cells or disseminating genetic contents toward distal organs [37–39].

Although therapeutic potential of MSCs has been proven in preclinical studies and clinical trials for myriad diseases, conventional MSC therapy has several critical limitations to overcome; since MSCs act as a “living material” derived from different individuals, quality control is one of the major hurdles for their therapeutic use. Isolation procedure, culture condition, storage methods, and administration of MSCs can significantly affect cell viability as well as efficacy, leading to high cost and low reproducibility [40, 41]. Further genetic modification can be applied to improve therapeutic potency but must be tightly monitored and regulated to prevent unexpected safety issues such as ectopic differentiation and tumor formation. In this aspect, several attempts have been made to apply EVs as cell-free therapeutic candidates since EVs seem to reflect biophysical characteristics of parent cells. It has been noted that positive impacts of MSCs tend to persist for a long time despite their rapid disappearance following *in vivo* administration [6]. In addition, conditioned media collected from MSC culture can reproduce some benefits of MSC-mediated immunosuppression [42, 43]. Therefore, it is widely accepted that MSCs provide protective paracrine effects, which are at least partially exerted by the secretion of EVs. Indeed, it has been reported that MSC-EVs contain various cytokines, growth factors, metabolites, and even microRNAs produced by MSC itself and, therefore, have similar anti-inflammatory and regenerative effects as MSCs. Since EVs are cell free, storage and handling procedure can be much cost effective and safety concerns regarding immunogenicity, tumorigenicity, and embolism formation after EV injection are negligible compared to MSCs [44, 45]. Due to their liposome-like simple biological structure, EVs are stable *in vivo* compared to other foreign particles. Moreover, it is relatively easy to modify and/or improve the EV contents and surface property for enhancing the therapeutic potential or for utilizing as a drug delivery system [46–48].

In this review, we will summarize and discuss the major studies investigating the efficacy of MSC-EVs in both *in vitro* and *in vivo* models mainly focusing on their immunomodulatory properties to provide up-to-date information in EVs and MSC therapeutic fields.

## 2. Immunomodulatory Efficacy of MSC-EVs in Animal Models of Immune Disorders

In a number of *in vivo* observations, therapeutic potential of MSC-EVs has been proven against various animal models of diseases accompanied by excessive inflammation (Table 1).

In a rat model of inflammatory bowel disease (IBD) induced by trinitrobenzene sulfonic acid (TNBS), intravenous injection of EVs derived from rat bone marrow MSCs improved the gross and histological symptoms of colitis, including body weight loss, disease activity index, destruction of the colonic structure, and immune cell infiltration via attenuation of colonic inflammation, oxidative stress, and apoptosis [49]. In a similar study, EVs derived from human umbilical cord MSCs ameliorated IBD symptoms in dextran sulfate sodium- (DSS-) induced colitic mice, presumably through the regulation of IL-7 production in macrophages. In addition, labelled EVs were detected in colon tissues of colitic mice at 12 hours after administration [50].

Cosenza et al. reported the therapeutic efficacy of MSC-EVs against inflammatory arthritis using murine models of delayed-type hypersensitivity (DTH) and collagen-induced arthritis (CIA). MSC-EVs exerted the dose-dependent anti-inflammatory effects in both models through the inhibition of B cell maturation, as well as the induction of regulatory B cells in lymph nodes [51]. In a similar study of porcine synovitis, EVs derived from porcine bone marrow ameliorated the symptoms of antigen-induced synovitis and reduced the inflammation in the joint. The decreased number of synovial lymphocytes was detected along with a downregulation of tumor necrosis factor- (TNF-) *α* transcripts within joints treated with EVs [52].

Sepsis, a systemic inflammatory response against microbial infection, is one of the targets for MSC-based therapy, because the mortality rate of sepsis remains high in intensive care units despite advanced development of antibiotics. Recently, MSC-EVs have been evaluated for their efficacy in rodent models of sepsis induced by cecal ligation. In a rat model of sepsis syndrome, treatment of adipose MSC-derived EVs alleviated the systemic inflammatory response, organ damage, and subsequent lethality. In the study, the potency of EVs derived from healthy (normal culture condition) or apoptotic (induced by hypoxia and serum starvation) MSCs was compared. Although no significant difference was observed in the mortality rate, healthy MSC-derived EVs more efficiently downregulated the levels of inflammatory mediators compared to apoptotic MSC-derived EVs [53]. In another study of sepsis by Song et al., EVs derived from MSCs pretreated with IL-1*β* exerted higher therapeutic efficacy against murine sepsis models than EVs from naïve MSCs. They demonstrated that EVs from primed MSCs effectively polarized macrophages into the M2 type, which is the anti-inflammatory phenotype of macrophages. Importantly, miR-146a, a well-known anti-inflammatory miRNA, was significantly upregulated in IL-1*β*-treated MSCs and packed into EVs. miR-146a packed in EVs was transferred into macrophages to polarize them into the M2 type [54].

In murine models of acute graft-versus-host disease (GVHD) induced by allogeneic transplantation of hematopoietic stem cells, EVs released from human umbilical cord MSCs (hUC-MSC-EVs) were assessed for prophylactic effects. hUC-MSC-EVs ameliorated the symptoms and histopathology of GVHD, leading to the increased survival rate. An absolute number of cytotoxic T cells were significantly decreased in the EV-treated group along with the downregulated serum levels of IL-2, TNF-*α*, and interferon- (IFN-) *γ*. On the contrary, the serum IL-10 level was elevated by EV treatment [55].

Type-1 diabetes mellitus (T1DM) is an autoimmune disorder leading to the irreversible destruction of insulin-producing cells in pancreatic islets. A recent study revealed that EVs derived from adipose tissue-derived MSCs can reduce clinical symptoms of streptozotocin- (STZ-) induced T1DM. Intraperitoneal injection of EVs into T1DM mice prevented hyperglycemia, body weight loss, lethality, and islet degeneration by STZ. Moreover, in splenocytes from EV-treated T1DM mice, levels of IL-17 and IFN-*γ* were significantly decreased whereas those of IL-4, IL-10, and TGF-*β* were increased along with the elevation in regulatory T (Treg) cell proportion [56]. Given that MSC-EVs can regulate excessive inflammation, these EVs can be utilized to support the stable transplantation of cells or organs, represented by islet transplantation. Wen et al. proved that human EVs can improve the efficiency of islet transplantation. EVs harvested from human bone marrow MSC and PBMC coculture improved the outcome of islet transplantation in humanized mouse models through the generation of Treg cells [57].

Since MSCs can accelerate the healing of tissues from injury or wound through the immunomodulatory function, several groups tried to investigate whether MSC-EVs could reproduce this ability. Li et al. demonstrated that EVs from human umbilical cord MSCs could attenuate excessive inflammation induced by burn injury. In the study, miR-181c in EVs was found to be critical for immunoregulation and EVs overexpressing miR-181c more efficiently reduced inflammation in burned rats [58]. In addition, MSC-EVs exhibited immunosuppressive effect against concanavalin A- (ConA-) induced liver injury models. The intravenously injected MSC-EVs were detected in the liver. While the aminotransferase (ALT) level, liver necrosis, and apoptosis were decreased, mRNA expression of anti-inflammatory cytokines and the number of Treg cells were increased [59]. In another very recent study, EVs from human umbilical cord-derived MSCs promoted locomotor functional recovery after spinal cord injury. EVs regulated the ratio of local M1/M2 subset macrophages in injured spinal cord and the production of macrophage-produced cytokines [60].

## 3. Mechanism of Immunomodulation by MSC-EVs

### 3.1. MSC-EVs and Macrophages

Macrophages, one of the principal components of the innate immune system, are originated from either yolk sac during embryonic development (tissue-resident macrophages) or bone marrow-derived monocytes (circulating macrophages) and involved in inflammatory response via phagocytosis and antigen presentation as well as in tissue homeostasis [61]. Upon activation, resting M0 macrophages are differentiated into classically activated M1 and the alternatively activated M2 phenotypes. In general, M1 macrophages secrete proinflammatory molecules including TNF-*α* and IL-1*β*, while M2 macrophages are regarded as anti-inflammatory cells producing immune-modulating factors such as IL-10 [62]. Since M1/M2 macrophages have distinct roles in both innate and adaptive immune systems, it is not surprising that disturbance of M1/M2 balance is often observed in various pathological conditions. Therefore, immunomodulatory effects of MSCs seem to be largely dependent on the regulation of abnormal macrophage activity [63, 64]. In recent years, MSC secretome analyses have shown that MSCs can produce various chemokines, growth factors, and other signaling molecules affecting polarization, maturation, proliferation, and migration of macrophage [65, 66] and growing evidence supports that MSC-EVs also recapitulate the beneficial effect of MSCs on macrophage regulation (Table 2). Shen et al. have reported that MSC-EVs could prevent renal dysfunction after ischemia-reperfusion injury [67]. They examined macrophagic infiltration in the kidney and found that MSC-EVs decreased recruitment of macrophage, implying that MSC-EVs impede chemotaxis of activated macrophage. It is noted that MSC-EVs express C-C motif chemokine receptor 2 (CCR2), a specific receptor for proinflammatory chemokine C-C motif chemokine ligand 2 (CCL2), compared to fibroblast EVs. *In vitro* migration assay proved that CCR2 of MSC-EVs acts as a scavenger for CCL2 and hence prevents macrophagic accumulation and further tissue damage. Indeed, EVs isolated from CCR2 siRNA-transfected MSCs failed to provide the protective effects of control siRNA EVs. Similarly, high levels of various chemokines such as chemokine (CXC motif) ligand 10 (CXCL10), monocyte chemoattractant protein 1 (MCP-1), CXCL9, and tissue inhibitor of metalloproteinase-1 (TIMP-1) led to an accumulation of cytotoxic macrophage in colitis-induced colons, while MSC-EVs could reduce these proinflammatory cytokines and macrophage-mediated tissue damage [68]. In addition, EV-derived microRNAs can enhance the M1 inhibitory ability of MSC-EV treatment in the context of aortic aneurysm formation after elastase infusion [69]. In this model, the severity of elastase-induced aortic damage correlated with proinflammatory cytokine levels. MSC-EVs successfully ameliorated aortic dilation and immune cell infiltration partly by downregulation of proinflammatory and chemokine signaling. Importantly, inhibition of M1 macrophage-derived cytokines including high-mobility group box 1 (HMGB1), chemokine (C-C motif) ligand 5 (CCL5), and macrophage inflammatory protein 1a (MIP1a) was miR-147 dependent regarding that the miR-147 inhibitor mitigated the beneficial effects of MSC-EVs.

In addition, the therapeutic capacity of MSC-EVs targeting imbalance of M1/M2 polarization has been proven in various disease models. In an experimental murine model of bronchopulmonary dysplasia (BPD), MSC-EVs ameliorated pulmonary fibrosis and the histological lung injury score by reducing hyperoxia-induced inflammation [70]. Authors found that MSC-EVs could mitigate several proinflammatory signals such as CCL5, TNF-*α*, and IL-6 from M1 macrophages while enhancing the M2 macrophage-derived immunomodulatory factor, Arginase 1 (Arg1). Others have shown that MSC-EVs can modulate tissue-specific macrophage polarization towards the tissue regenerative/repair phenotype. Notably, *in vivo* tracking data of fluorescence-labelled MSC-EVs after intravenous injection suggested that EVs have homing capacity to the injury site as MSC itself and macrophages, especially M2 types, are the primary target cell of EV localization in the damaged spinal cord [71]. Using a high-fat diet-induced obesity model, Zhao et al. proved the therapeutic effect of MSC-EVs on metabolic dysfunction and chronic inflammation within the white adipose tissue via EV-educated macrophages. They found that MSC-EV uptake by macrophages resulted in M2 polarization through the EV delivery of the activated signal transducer and activator of transcription 3 (STAT3) protein which in turn upregulated Arg1 expression of macrophages [72].

Since EVs contain various bioactive molecules including peptides, lipids, and nucleotides, preconditioning of MSC with inflammatory stimuli could contribute to generating more immunoreactive EVs. Ti et al. reported that EVs produced by lipopolysaccharide- (LPS-) treated MSC (LPS-pre-EVs) exhibited more potent M2 induction capacity than those from control MSCs [73]. Interestingly, LPS-pre-EVs expressed a stable level of microRNA Let-7b, which can impede TLR-4 signaling thereby inducing M2 polarization followed by nuclear factor-*κ*B (NF-*κ*B) inhibition. On the contrary, EV-derived Let-7b activated the STAT3 pathway, one of the transcriptional repressors of inflammatory signaling, as well as survival-related Akt signaling. Overall, LPS-pre-EVs could accelerate wound healing during diabetes compared to MSC-EVs. In another report, MSCs under hypoxic culture condition were found to secrete EVs containing wound healing process-related microRNAs such as miR-223, miR-146b, miR-126, and miR-199a and these EVs readily correct the M1/M2 balance in muscle injury models [74]. Finally, genetic manipulation of MSCs can enhance the therapeutic capacity of EVs. Jiang et al. have evaluated the benefits of miR-30d-5p, known as an autophagic suppressor, in brain injury models based on the finding that the serum level of this microRNA was significantly decreased in stroke patients [75]. To harvest therapeutic factor-enriched EVs, they genetically modified MSCs to produce the extra level of miR-30d-5p. It is noted that overexpressed miR-30d-5p accumulated within the EVs and enhanced M2 microglial polarization via Beclin-1 and atg5 inhibition, leading to amelioration of cerebral damage. These observations suggest that both naïve and engineered MSC-EVs can not only modulate macrophagic activity to resolve excessive inflammation but also stimulate tissue repair/regeneration.

### 3.2. MSC-EVs and Other Types of Immune Cells

A growing number of studies suggest that other effector cells of innate and adaptive immune systems could be regulated by MSC-EVs. Similar to macrophages, dendritic cells (DCs) can function as antigen-presenting cells and bridge innate to adaptive immune systems. Interestingly, several reports have already demonstrated that MSCs have suppressive roles in DC activation in a secretory factor-dependent manner, implying that MSC-EVs itself could regulate the fate of DCs [76–78]. Indeed, when MSC-EVs were treated to DCs derived from patients with type I diabetes, mature DC markers such as CD80, CD86, CCR7 receptor, and HLA II molecules were significantly decreased compared to those of vehicle-treated DCs [79]. Moreover, these MSC-EV-stimulated immature DCs produced immunomodulatory factors, TGF-*β* and PGE2, leading to an induction of regulatory T cells during DC and naïve T cell coculture.

In a rat experimental autoimmune uveitis (EAU) model, periocular injection of MSC-EVs restored EAU damage and retinal functions by reducing CD161^+^ NK cell trafficking within the lesions, although the exact contributing EV factors underlying these therapeutic outcomes have not been defined yet [80, 81]. In addition, Di Trapani et al. have described that EVs derived from IFN-*γ*- and TNF-*α*-primed MSCs were localized in CD19^+^ B cells and CD56^+^ NK cells as well as CD3^+^ T cells and exhibited some immunosuppressive effects by miR-155- and miR-146-dependent inhibition of cell proliferation [82]. The immunomodulatory impact of MSC-EVs on B cell function has been also proven by others using CpG-induced B cell stimulation assay. They found that MSC-EVs could inhibit both proliferation and maturation of B cells, leading to a decrease in secretion of immunoglobulin [83].

A large body of studies have shown that the immunomodulatory action of MSCs is partially mediated by the suppression of proliferation, differentiation, and activation of T lymphocytes [10, 84]. This T cell-modulating ability of MSC-EVs has been demonstrated in several *in vitro* and *in vivo* experiments. Blazquez et al. reported that EVs from human adipose tissue-derived MSCs can suppress the proliferation of T cells [85]. Moreover, Amarnath et al. revealed that a possible mechanism of T cell modulation by MSC-EVs could involve adenosine A2A receptors [86]. Another study from Del Fattore et al. also demonstrated that EVs from human bone marrow MSCs increased the ratio of regulatory T cells compared to effector T cells along with the increase in the IL-10 level [87]. In addition, several studies determined the in vivo generation of regulatory T cells in different disease models. Zhang et al. showed that EVs from human embryonic stem cell-derived MSCs could induce the generation of regulatory T cells in allogeneic skin graft models [88]. In murine models of liver injury or islet transplantation, MSC-EVs were found to induce regulatory T cell generation [57, 59]. Although these interesting studies have reported the immunomodulatory functions of MSC-EVs on T cell activity, there are also controversial opinions reporting that the immunomodulatory effects of MSC-EVs on T cells were minimum or lower compared with MSCs themselves [89, 90]. Conforti et al. have shown that PBMC-derived T cell proliferation induced by phytohemagglutinin treatment was significantly reduced by MSC coculture in a cell number-dependent manner, while MSC-EVs had little but no significant impact. Analysis of PBMC and MSC- or MSC-EV-co-cultured supernatant revealed that immunomodulatory molecules such as IL-10 and PGE_2_ were abundant in the supernatant with MSC compared to MSC-EVs [90]. In addition, Del Fattore et al. reported that MSC-EVs could increase not only proliferative but also apoptotic Treg cells following CD3/CD28 stimulation, while MSCs did not affect T cell death [87]. These data imply that the immune regulatory ability of MSC and MSC-EVs might vary depending on the context and should be carefully evaluated to optimize their therapeutic potential.

### 3.3. Clinical Application of Exosomes and Future Direction/Limitation

So far, two clinical studies of MSC-EVs have been performed. In the study by Kordelas et al. [91], a patient with steroid refractory graft-versus-host disease (GvHD) was administered with allogeneic MSC-derived EVs. Before the administration into the patient, *in vitro* analysis for the evaluation of MSC-EVs was performed. In mixed leukocyte reaction using patient-derived peripheral blood mononuclear cells (PBMCs), MSC-EVs exhibited the suppressive effect on the proliferation of PBMCs secreting proinflammatory cytokines, including IL-1, TNF-*α*, and IFN-*γ*. Moreover, MSC-EV treatment in the patient resulted in significant improvement of GvHD symptoms for more than four months. In another study, the therapeutic effect of MSC-EVs was investigated in forty patients with chronic kidney disease (CKD) [92]. MSC-EVs were intravenously infused, followed by the second treatment though an intra-arterial route with a one-week interval. Adverse events were not observed, and MSC-EV-treated patients exhibited significant improvement in kidney functions evaluated by the glomerular filtration rate, urinary albumin/creatinine ratio, blood urea level, and serum creatinine level, compared to the placebo group. Moreover, levels of TGF-*β* and IL-10 in peripheral blood were increased at 12 weeks and even 1 year after MSC-EV treatment, whereas the level of TNF-*α* was decreased.

These two clinical studies propose MSC-EVs as promising immunomodulatory therapeutics; however, the following challenges should be considered for the practical application of EVs. First of all, acquiring large scales of MSC-EVs with high purity would be a main issue in this field. Since MSC-EVs are isolated from MSC culture media, culture conditions including the seeding cell number, media volume, and EV harvest timing can influence both the quantity and quality of EVs. In addition, the most effective EV isolation method from culture media has not been established yet. Therefore, optimization of culture methods (e.g., hypoxia, sheer stress, and bioreactor) combining with intensive evaluation of the pros and cons of the different EV isolation methods should be preceded to improve the yield of MSC-EVs and these procedures should be regulated and controlled to ensure the clinical-grade exosome production. Recently, Mendt et al. evaluated the therapeutic effects of BM-MSCs on pancreatic cancer xenograft mouse models to provide feasible directions for clinical application of MSC-EVs [44]. In this report, BM-MSCs were cultured using a bioreactor system in the GMP facility to obtain sterile, clinical-grade EVs. *In vivo* distribution analysis of fluorescence-labelled EVs has shown that MSC-EVs might have homing capacity to the injured or tumor-bearing site as MSCs. They also evaluated the long-term toxicity and immunogenicity of repetitive EV administration using hematological examination, histopathological analysis, and immunotyping test, which all supported that MSC-EVs might not trigger any immune response or toxic reaction. Further preclinical and clinical evaluation of EVs in various disease conditions should be followed to ensure the safety and efficacy of MSC-EVs.

Since MSC-EVs theoretically contain various MSC-derived bioactive molecules, precise mechanisms of action or key therapeutic factors have not been disclosed. To define the key factors, comparative transcriptome/proteome analysis of MSC-EVs has been conducted and revealed their differential properties in terms of functional enrichment of gene analysis and microRNA expression patterns [93, 94]. These results imply that big data-based analysis of transcriptome and proteome enables us not only to understand the common nature of MSC-EVs but also to compare unique characteristics and advantages by their origins, contributing to the application of optimized MSC-EVs for appropriate target disease.

## 4. Conclusions

A number of most recent experimental evidences suggest that MSC-derived EVs might carry similar immunomodulatory properties of MSCs, which could be beneficial for the treatment of inflammatory diseases via direct immunosuppressive function, as well as for the regenerative purpose through the improvement of the inflammatory niche. As discussed in this review, EVs from human or animal MSCs mostly contributed to the attenuation of excessive inflammation to alleviate the symptoms of immune disorders or improve the efficiency of allogeneic transplantation. Because EVs possess valuable advantages in that they can overcome the reported limitations of parental cells, including safety, reproducibility, and cost effectiveness related with storage and maintenance, there is no doubt that MSC-EVs might be a novel promising therapeutics. However, although EV's modes of action in macrophage polarization and B/NK/T cell suppression have been reported as in their parental cells, a variety of further investigations are required to precisely elucidate their mechanisms regarding immunosuppression or tolerance induction, specific for each disease condition. Furthermore, the standardization and optimization of EV production should be established along with the investigation of their efficacy and underlying mechanisms to resolve the current hurdle in the development of EV-based therapy.

## Figures and Tables

**Table 1:** Effects of MSCs on experimental animal models of inflammatory conditions.

Model	Animals (strain)	MSCs			Ref.
		Source	Route	Effects & note	
IBD (TNBS induced)	Rat (SD)	Rat BM	IV	Suppression of inflammation, oxidative stress, and apoptosis in colon tissues	[49]
IBD (DSS induced)	Mouse (KM)	Human UC	IV	Regulation of IL-7 production in macrophages	[50]
Arthritis (DTH)	Mouse (BALB/c)	Mouse BM	Footpad	Anti-inflammatory effects through the suppression of plasmablast differentiation and generation of Breg cells	[51]
Arthritis (CIA)	Mouse (DBA/1)	Mouse BM	IV		[51]
Synovitis	Pig (white pig)	Pig BM	Intra-articular	Decreased synovial lymphocytes and downregulation of TNF-*α* transcripts	[52]
Sepsis (cecal ligation)	Rat (SD)	Rat AT	IV	Decreased levels of inflammatory mediators in circulation, bronchioalveolar lavage, and abdominal ascites	[53]
	Mouse (C57BL/6)	Human UC	IV	Reduction of inflammation and lethality through the regulation of macrophage polarization	[54]
GVHD (allo-HSCT)	Mouse (BALB/c)	Human UC	IV	Suppression of cytotoxic T cells and inflammatory cytokine production	[55]
T1DM (STZ induced)	Mouse (C57BL/6)	Mouse AT	IP	Symptom reduction via regulation of Th cell subtype differentiation	[56]
Islet transplantation	Mouse (NSG)	Human BM	IV	Support stable transplantation of islet via Treg cell induction	[57]
Burn injury	Rat (SD)	Human UC	IV	Attenuation of excessive inflammation by miR-181c	[58]
Liver injury (ConA induced)	Mouse (C57BL/6)	Mouse BM	IV	Decrease in ALT, liver necrosis, and apoptosis via Treg cell generation	[59]
Spinal cord injury	Mouse (C57BL/6)	Human UC	IV	Functional recovery of spinal cord injury through downregulation of inflammatory cytokines	[60]

IBD: inflammatory bowel disease; TNBS: trinitrobenzene sulfonic acid; DTH: delayed-type hypersensitivity; CIA: collagen-induced arthritis; GVHS: graft-versus-host disease; allo-HSCT: allogeneic hematopoietic stem cell transplantation; T1 DM: type 1 diabetes mellitus; STZ: streptozotocin; ConA: concanavalin A; BM: bone marrow; UC: umbilical cord; AT: adipose tissue; IV: intravenous; IP: intraperitoneal; Breg: regulatory B cells; TGF-*β*1: transforming growth factor beta 1; Th cell: helper T cell; Treg cell: regulatory T cell; ALT: aminotransferase.

**Table 2:** Regulatory mechanisms of MSC-EVs on macrophage polarization.

EV source	Disease model	Effects	Defined key factors in EVs	Ref.
Mouse BM-MSCs	Renal injury	Chemotaxis inhibition M1 suppression	CCR2	[67]
Human UC-MSCs	Inflammatory bowel disease	M1 suppression M2 induction	NA	[68]
Human UC-MSCs	Abdominal aortic aneurysm	M1 suppression	miR-147	[69]
Human BM-MSCs	Bronchopulmonary dysplasia	M1 suppression M2 induction	NA	[70]
Mouse BM-MSCs	Spinal cord injury	M2 induction	NA	[71]
Mouse AT-MSCs	Obesity-induced inflammation	M2 induction	Activated STAT3	[72]
Human UC-MSCs	Diabetic cutaneous wound	M2 induction	Let-7b	[73]
Human AT-MSCs	Muscle injury	M2 induction	miR-223, miR-146b, miR-126, and miR-199a	[74]
Human AT-MSCs	Ischemic brain injury	Microglial M2 induction	miR-30d-5p	[75]

BM: bone marrow; UC: umbilical cord; AT: adipose tissue; CCR2: C-C chemokine receptor type 2.
